# Demographic characterization and social patterns of the Neotropical pampas deer

**DOI:** 10.1186/2193-1801-2-259

**Published:** 2013-06-10

**Authors:** Mariana Cosse, Susana González

**Affiliations:** Departamento de Genética, IIBCE-Facultad de Ciencias/UdelaR, Genética de la Conservación, Av. Italia 3318, 11600 Montevideo, Uruguay

**Keywords:** *Ozotoceros bezoarticus*, Population dynamics, Sexual aggregation-segregation pattern, Ungulates, Conservation, Uruguay

## Abstract

The most endangered subspecies of pampas deer *Ozotoceros bezoarticus uruguayensis* is an endemic cervidae of the Uruguayan temperate grasslands. The aim of our study was to assess the demographic trends, grouping structure and dynamic of this small and isolated population. We surveyed the population during seven years and detected an average of 117 (+ 72.7 SD) individuals (44 censuses). The average population structure observed was 55% adult females, 34% adult males, 10% juveniles, and 1% fawns, with a low recruitment rate of 0.11. The pampas deer is a gregarious cervidae with 62% of individuals being observed within groups of at least three animals. Nevertheless we observed substantial differences on group size and composition based on sex, reproductive status, season and trophic resources availability. The population dynamics showed significant changes around the year in the sexual aggregation-segregation pattern, corresponding with reproductive and physiological status. The mean density on this population (11 deer/ km^2^) is the highest reported for the species. Comparable data, from other populations, showed a significant correlation between density and sex ratio, with a reduction in the proportion of males with higher deer densities. An action plan for this endangered population should include initiatives involving private landowners, and guidelines to improve the deer habitat.

## Introduction

The pampas deer, *Ozotoceros bezoarticus* (Linnaeus 1758), is an ungulate native to the open grassland habitats of South America, from 5° to 41°S (Cabrera, 1943;Jackson & Langguth, 1987, González et al.*,*^2010^). Over the last two centuries its natural habitats (the Pampas, Cerrado, and grasslands of Argentina, Uruguay, Paraguay, Bolivia, and southern Brazil) have been drastically modified by agricultural activities (Fonseca et al., 1999;González et al., 2010). The pampas deer have been deeply affected by habitat alteration and fragmentation, which are considered to be one of the main causes of the reduction and populations decline in the species’ range (González et al., 1998). This process is clearly visible in Uruguay, which is home to two endemic subspecies: *O. b. arerunguaensis*, represented by a population of around 1000 individuals in El Tapado (northern Uruguay); and *O. b. uruguayensis*, with less than 350 individuals, located at Los Ajos in southeast Uruguay (González et al., 2002). While populations of the other subspecies (*O. b. leucogaster, O. b. bezoarticus*, and *O. b. celer*) are found in protected areas in Argentina, Bolivia, and Brazil, the Uruguayan populations are located exclusively on private land where several agricultural activities are conducted (Jackson et al., 1980, Cosse et al., 2009;González et al., 2010).

An understanding of the spatial relationships between species and their environments is essential to prioritize research and conservation needs. Group structure is shaped by feeding and anti-predatory strategies (Caughley, 1964). Group foraging generally entails costs and benefits for the individuals, associated with the presence of other individuals of the same species (Krause & Ruxton, 2002). Determining a population’s grouping strategy and its variation in different environments can provide information on various aspects, such as quality of the environment and hunting pressure (Morrison et al., 2006).

In most social ungulate species, males and females live in separate groups outside the breeding season, usually using different home ranges, or different kinds of habitats (Clutton-Brock et al., 1982). Ruckstuhl (2007) provides a comprehensive review of the hypotheses proposed to explain sexual segregation among ungulates. Comprehension these processes in pampas deer is key for understanding the mechanisms that shape the genetic structure of the populations and the implications of the habitat they occupy for the degree of genetic variability and management options.

Previous observations made in several populations showed pampas deer living in small herds, with a group size of one to twelve, but rarely more than five or six animals (Fitzroy & Darwin, 1839, Cabrera et al., 1940;Merino & Beccaceci, 1999, Eisenberg, 2000;Mourão et al., 2000, Dellafiore et al., 2003, Pereira et al., 2005). According to Jackson & Langguth (1987) wild herds are dynamic in size and composition. Especially the males, move from one group to another, but mother-young bonds appear to be strong with fawns staying with their mother until they are at least one year old. However the dynamics of the Pampas deer group composition in agro-ecosystems has not been systematically studied.

The aim of our study was to document the group structure, demography and sex segregation behavior on an isolated pampas deer population in Uruguay. We estimated the population’s size to evaluate the population trend; and we assessed the seasonal variations on group’s conformation. Furthermore, we examined whether mean group size was linked to population density. At the same time, we determined if there are differences in the type of group (nursery/bachelor groups or mixed group) chosen by an individual deer based on deer sex or reproductive status. We examined whether sexual segregation and aggregation within the population was related to life cycle.

## Methods

### Study area and animals

The study area is mainly located in an 80 km^2^ ranch (33° 50′ 01″ S; 54° 01′ 34″ W) within the “Bañados del Este” Biosphere Reserve at Ramsar Area, in the southeastern Uruguayan Department of Rocha. The landscape is characterized by low, rolling hills; the parent material consists of Quaternary unconsolidated sediments (clays, argillaceous mud, and sands and, locally in rocky points, igneous or metamorphic rocks). The soils are predominantly gley soils. Altitudes range from −5 to 100 masl, the annual average rainfall is 1000 mm and the average annual temperature is 16°C (Cosse et al., 2009). The main land use activities are livestock (cattle and sheep) ranching, agricultural crops (rice, soy, wheat) and pasture (ryegrass). The pampas deer are free ranging in this complex landscape of discrete patches (paddocks) and are conspicuously patchy in their distribution among paddocks. The paddocks vary on grass species and livestock load.

### Data recording

We conducted fieldwork seasonally over the course of seven years, from 1996 to 1999 and 2002 to 2004. Both surveyed periods were assembled previously we performed a Homogeneity of Variances Analysis to perform the demographic calculations.

Observations were made early in the morning from a vehicle traveling at a slow constant speed through a fixed 8 km route, with surveys averaging three hours in duration We performed the observations using a 10 × 40 telescope and at distances of up to 500 m. We estimated Herd Composition Count (HCC) (Kaji et al., 2005). For each survey, we recorded the mean group size and group composition. We defined the groups as singletons (adult male, adult female), bachelor groups (only males), nursery groups (including females and sub-adults/juveniles of unknown sex) and mixed-sex groups. The two sexes were identified based on the presence or absence of antlers, and the year’s offspring were distinguished from older animals on the basis of body size. In cases where sex or group composition could not be determined reliably, data was excluded from the analysis. Monthly observations were pooled as seasonal observations as follows: 1: summer; 2: autumn; 3: winter and 4: spring.

We estimated the typical group size (TGS) following Jarman (1974) and Moore (2001):

Where n_i_ is the number in each group_i_ and N is the total number of groups observed (Jarman, 1974). This parameter provided information on the size of the group observed for an average individual.

In the mixed-sex groups with three or more individuals (with no unidentified individuals) the sex ratio (number of males/number of females) and juvenile ratio (number of juveniles/total group size) were determined. For each census we analyzed the proportion of each category of individuals in every group type. We used the Kruskal–Wallis (KW) variance analysis to test the differences between variables (Zar, 1999). We run an *r x c* contingency test (Milton & Tsokos, 1987) to analyze the difference in the type of association (singletons, mixed-sex groups, single-sex groups) presented by individuals based on their sex. The contingency table was run on online software Statistics to Use (Kirkman, 1996).

### Density

The pampas deer in Los Ajos are conspicuously patchy in their distribution among paddocks. We estimate two densities values: i) the mean global density, corresponding with the number of deer surveyed on total area they occupy; ii) the mean density only on occupied paddocks, to estimate the deer load on the pastures we calculated the deer density based on the number of individuals per paddock (area) for each survey. We correlated the mean group size and density per paddock and year.

### Population viability analysis

We examined the complex interactions between pampas deer demography, environmental and genetic factors by computer simulation modeling, using the program VORTEX version 9.5. VORTEX has been widely used in several endangered species and is especially powerful for modeling vertebrate wildlife population behavior (Lacy et al. 2009).

The program yields the following information: probability of persistence of the population, for a maximum population size (Nmax); the expected persistence time, will depend on the average growth rate *r* (r = nº births – nº deaths) as well as the variance of this parameter due to environmental fluctuations; size of the average population along time; expected and observed average heterozygosity; average number of alleles; final allele composition of the population; population growth rate (stochastic and deterministic r); probability of extinction is defined as the absence of any of the sexes.

We considered the data from the population structure observed in the surveyed years (sex ratio and age structure), and also the data of the sample of animals captured (González & Duarte, 2003). The variables used were: size of the initial population, one basic scenario was performed, with an initial population size 400, based in current data; for the 400 individual population, age and sex classes were calculated in proportion to the real distribution; during simulations VORTEX distributes the age-sex structure according to the reproduction and death rates specified in each scenario, using the deterministic algorithms of Leslies’ matrix. We run 200 iterations, for 350 years. Results were summarized every ten years.

One basic scenario was considered, with a carrying capacity of the environment (K) of 600 (s.d. 50) individuals. VORTEX fixes carrying capacity as an upper limit for population size, beyond which an additional mortality rate is imposed, proportional along all age-sex classes in order to return the population to the specified K value. Migration and supplementation were not considered. Reproductive system variables considered: polygyny mating system; 18 month of age of reproductive maturity of females and 2 years of males; maximum breeding age (senescence) of 6 years for both sexes; sex ratio at birth 50%; average litter size of 1; proportion of adult females in the breeding pool per year was of 85% (s.d.: 5%) and for adult males was 90% and successfully siring 72%; non inbreeding was considered in the modeling.

The annual mortality rate of individuals under 1 year old of age for females was considered: 32% (s.d. = 5%), and for males 40% (s.d. = 5%) as a maximum value related to environmental conditions, being the first year of life the most critical in large mammals. The annual mortality rate of animals above 1 year of age is 10% (s.d. = 5%).

### Sexual segregation

We analyzed the sexual segregation or aggregation pattern using the SSAS index (Bonenfant et al., 2007). We conducted the analyses using the R program (R Development Core Team, 2009) following the protocol developed by Bonenfant et al. (2007).

### Inter-population analysis

We compared the mean group size and density reported in the literature from different populations in the species’ distribution range, including information on sex ratio, latitude and longitude. A multiple regression analysis was performed to determine the best predictors of group size and the association between different variables (Zar, 1999).

## Results

### Demographic parameters

We conducted 44 surveys over seven years, and analyzed a total of 2149 groups. The mean population size estimation was 117 (SD = 72.7). The average ratio of adult males to adult females was 0.61. This sex ratio deviated significantly from a 1:1 ratio (χ2 = 167.5, df = 1, p < 0.05).

The overall population structure observed was 55% adult females, 34% adult males, 10% juveniles, and 1% young. The demographic parameters estimated were: mean size of the population ; sex and age structure: 40 ♂♂:64♀♀: 12juv.: 1fawn; mean recruitment rate ; age of first reproduction; 1.5 years; litter size: 1 fawn/♀.

### Social structure and groupings

The mean size for the 2149 groups observed was 2.4 individuals (range 1–67; 3.56 SD) and the mean Typical Group Size in 44 censuses was 7.18 (range 1.9 – 37.9; 7.9 SD). We did not obtain a significant correlation between mean group sizes or mean Typical Group Size and density per enclosure, neither overall or seasonally.

When we analyzed the average annual group compositions we found that, 19% of the groups were singletons, 19% were in couples, 44% were groups of three to nine animals, and 18% were in groups of 10 or more individuals.

**Figure 1:**
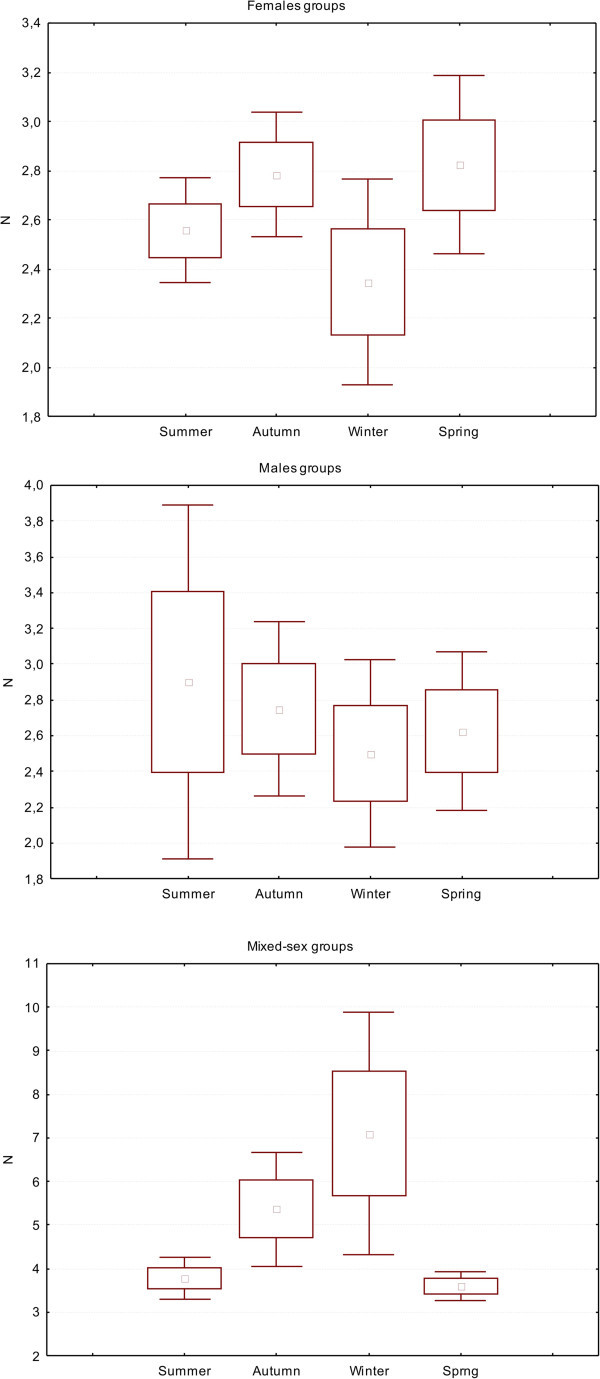
**Mean group size (□) of females (upper), males (middle) and mixed-sex (bottom) in the different seasons.** Mean ± SE (□) and mean ± 1,96*SE(⊤).

We recorded 175 nursery groups, with a mean 2.7 individuals (range 2–7; 1.1 SD). We found a significant variation in the size of these groups based on the season (KW: GL = 3; N = 175; H = 11.02; p = 0.01), whose size dropped to a minimum during birth season. In contrast, we observed no significant seasonal variation in the size of the bachelors groups (N = 66; mean size = 2.6, range 2–7, 1.2 SD). For mixed-sex groups (N = 391) the mean size was 4.70 (range 2–67; 6.2 SD), with larger groups observed in July and August (winter) (KW: GL = 9, N = 391; H = 25.08 p = 0.0029) (Figure 1).

**Figure 2:**
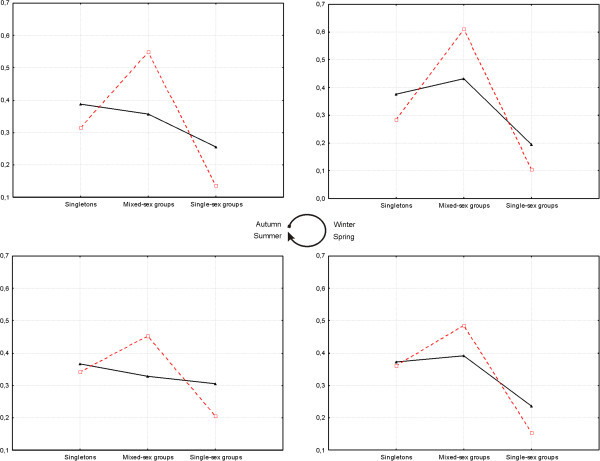
**Proportion of males (****) and females (▲) in the different group types (singletons; mixed-sex; single-sex).** Quadrants by season; Upper left: Autumn; Upper right: Winter; Lower right: Spring; Lower left: Summer.

The differences in the males and females distribution on different group type (singletons; mixed-sex groups; nursery or bachelors groups) based on sex, were statistically significant (χ2 = 38.5; df = 2; p < 0.0001, Table 1). Overall data showed similar proportions between males and females on singletons (32% of males was singletons and 35% of females); interestingly males proportions on mixed-sex groups are higher than females, with 53% of males conforming mixed-sex groups, respect to 42% on females. In this pampas deer population, the lower proportion of individuals were on single-sex group, only 15% of the males were on bachelors groups and 23% of the females were on nursery groups respectively; these proportions change around year but in all seasons males are in higher proportions than females on mixed-sex groups. On summer most females are on singletons and their proportion on mixed-sex groups and nursery groups are similar (32 and 30 percent respectively) (Figure 2).

**Table 1:** **Total numbers of females and males in the different type groups on the 44 population size estimations on seven years: single-sex groups refer to bachelor groups (males) and nursery groups (females)**

Individuals	Females	Males
Singletons	640	361
Mixed-sex groups	778	598
Single-sex groups	420	173
Total	1838	1132

### Los Ajos population density

The density data were obtained from 28 censuses conducted from 1998 to 2004, which provided information on the distribution of individuals in the various areas and paddocks. A total of 3290 individuals, forming 1645 groups, were recorded for this analysis. The area used by the deer was estimated to be 33.97 km^2^. We did not obtain significant differences in density by years or per enclosure, neither overall or seasonally. We calculated the mean global density as 3.46 (2.16 SD), however the mean pampas deer load per paddock was 11 deer per km^2^ (range 1.52 – 54.30; 0.98 SD) in 110 counts.

### Los Ajos population viability analysis (PVA)

The PVA showed the average population size maintain in the simulations in 291 individuals in the modeling scenarios. The probability of extinction of the population in simulated 350 years was very low 0.015. In all cases the population size increased rapidly until maximum carrying capacity is reached, in less than ten years. In our analysis the observed growth rate (r), r deterministic of 0.043 and r stochastic of 0.014 (SD = 0.061), being always very low but positive, and was mostly affected by the high mortality rates occurred during the first year of life.

### Sexual segregation-aggregation analysis

**Figure 3:**
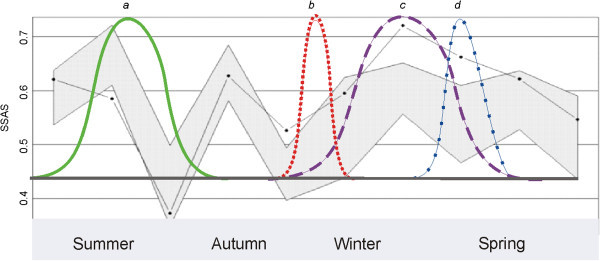
**Results of the SASS analysis overlaying events of the biological cycle of the Pampas deer.** The SSAS indicates significant sexual segregation or aggregation if the observed value (dotted line) falls above or below the shaded area (at the 5% error level), respectively. The seasons are represented on the axial axis. The graph show the annual biology cycle of Los Ajos Pampas deer: **a**) unbroken curve corresponds to the mating period; **b**) dotted curve covers the antler shedding period; **c**) broken lines represent the population’s birth curve; and **d**) broken dotted line represents the antler growth period.

We detected two annual periods with a non-random male–female association pattern with SSAS. In March (autumn) a sexual aggregation pattern was observed, and in September and October (spring) we observed a period of sexual segregation (Figure 3).

### Inter-populations analysis

**Figure 4:**
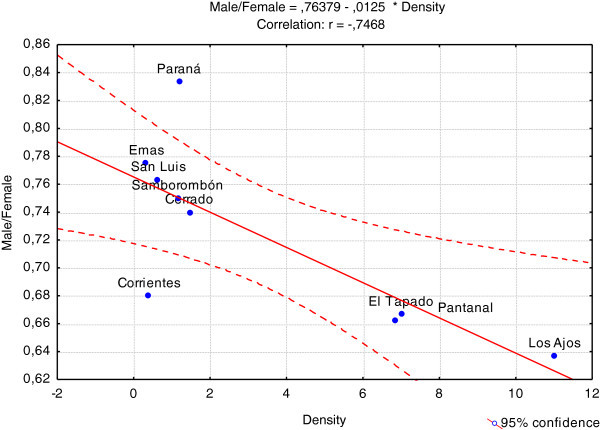
**Correlation graphs of sex ratio (male/female) and density (n°deer/km**^**2**^**) for populations on the distribution range of the species.**

We have performed comparative analyses including information for group size (mean value), density, sex ratio, latitude and longitude on different populations in Argentina, Brazil, and Uruguay (Table 2). We observed a significant correlation between sex ratio (M/F) and density (N = 9; Spearman = −0.76; t(N-2) = −3.11; p = 0.017) (Figure 4).

**Table 2:** **Mean group size, density in km**^**2**^**, and sex ratio (males/females) data for different pampas deer populations**

Population	Author	Latitude	Longitude	Average group size	Density	Male/female
Cerrado	Leeuwenberg & Lara-Resende (1994)	15.56	53.07	1.84	1.46	0.74
Emas	Redford (1987)	18.15	52.53	1.36	0.32	0.78
Pantanal	Lacerda (2008)	19.57	56.25	2.13	6.85	0.66
Paraná	Braga & Kuniyoshi (2010)	25.34	49.49	2.29	1.19	0.83
Corrientes	Merino & Beccaceci (1999)	28.08	56.33	1.75	0.39	0.68
El Tapado	Moore (2001)	31.36	56.43	2.20	7.00	0.67
Los Ajos	Cosse et al., present work	33.50	54.01	2.40	11.00	0.64
San Luis	Dellafiore et al. (2003)	34.22	65.44	-	0.63	0.76
Samborombón	Giménez Dixon (1991)	36.22	56.52	2.60	1.15	0.75

The geographic location of each population (latitude-longitude) is also indicated.

## Discussion

### Demographic parameters

Our mean size for the population estimation was 117 individuals, being very small and having along the study period remarkable fluctuations (range 16–254; 73 SD). Furthermore the biological examination of the captured specimens (González & Duarte, 2003) enabled to obtain valuable information in this pampas deer wildlife sample. We observed seven-year survival for the oldest male. Additionally seven from the eight females captured, were reproductively active (being pregnant or lactating, including the youngest at 18 months of age) representing 85% of the sample. The oldest female recorded in the capture had five years old and was lactating.

The parameters obtained in the PVA showed that the population is safe from the genetic and demographic factors for the next 350 years. In all the simulations we obtained very low and positive values of r, which shows that the population has a very low intrinsic growth potential. The carrying capacity of the environment is the main limiting factor. Increasing the habitat available will assure the persistence and expansion of the pampas deer.

This type of population structure is characteristic of “slow” life history, where the number of individuals is close to the carrying capacity, there is a strong resource selection, and a long life expectancy (Reznick et al., 2002;Jeschke & Kokko, 2009). Thus, population size seems to be stabilized by a structure of adult individuals and a failure to recruit young individuals. Our results were similar to those found in the Brazilian National Park Emas population, where it was estimated that adults represented 85 to 97.7% (Schaller & Duplaix-Hall, 1975, Redford, 1987). The low recruitment of young individuals was reported to El Tapado population, which was establish to have an estimated mortality rate of 0.4 for individuals under two years of age (Moore, 2001).

We estimated the sex ratio population as 0.61 (1 adult male to 1.63 adult female). The birth sex ratio at Los Ajos is unknown; in captivity a 1:1 sex ratio has been recorded (Ungerfeld et al., 2008). Nevertheless these results are not generalized to the Los Ajos population, since in some ungulate species in the wild, a relationship between readily-available resources and skewed fawns sex ratio has been observed (Kruuk et al., 1999, Bonenfant et al., 2003). Also to explain the skewed sex ratio in populations it is important to consider that in other Cervidae species is common young males disperse (a behavior that entails a high energy cost and greater mortality) while females exhibited a philopatric behavior (Coulon et al., 2006, Loe et al., 2009;Clutton-Brock & Lukas, 2012). Another explanation to this skewed ratio can be related with the agonistic behavior of males, which may increase the physiological stress (Loison et al., 1999, Pereira et al., 2006), generating individuals more sensitive to pathogens. Moreover, we observed on pampas deer populations a decline in the males proportions when population density rises (Table 2 and Figure 4). This pattern could correspond to a process where the females’ density is correlated with environmental carrying capacity, while the presence of males is determined by territoriality associated with resource defense polygyny tactic (Emlen & Oring, 1977;Vanpé et al., 2009). This tactic has been observed for various ungulate species (Dubost, 1970, Miura, 1984, Putman, 1988;Wahlstrröm, 1994). Nevertheless, the analysis in fecal testosterone demonstrated no significant differences in fecal testosterone concentrations among males from groups of varying sizes (Pereira et al., 2005). However, it will be need to assess the influence of resources on female distribution and their impact on male distribution and reproductive success (Vanpé et al., 2009).

### Social structure

Our observations confirmed that the pampas deer is a gregarious species with 62% of the individuals in Los Ajos population within groups of at least three animals.

However the grouping structure is variable and related with several factors. The most common type of groupings we observed in the Los Ajos population were basic units of two to four individuals (41% of the individuals) that appear to come together as feeding groups, with the largest groups forming in autumn or winter. These observations are in accordance with Pereira et al., (2005), who found large aggregations within Emas National Park population in feeding grounds such as burnt patches.

The mean group size values we observed for Los Ajos was 2.4, similar data were reported in other pampas deer populations (Redford, 1987;Merino & Beccaceci, 1999, Netto et al., 2000, Moore, 2001, Dellafiore et al., 2003, Pereira et al., 2005, Lacerda, 2008;Pérez Carusi et al., 2009;Braga & Kuniyoshi, 2010). The Typical Group Size (TGS) we observed at Los Ajos was 7.1 individuals, similar to the TGS (7.2 individuals) reported for the other Uruguayan population on Salto (Moore, 2001). The Uruguayan populations showed larger TGS than the recorded on Pantanal of 3.11 individuals (Lacerda, 2008). The differences between them may be due to the interaction of several factors, as carrying capacity, and including the behavior in different environments. The Uruguayan populations are mainly found in open grasslands and the Pantanal is a complex environment of open wet meadows and forested areas. Pereira et al., (2005) suggested that the low level of aggregation observed at cerrado biome is related to group instability and low density, characteristics that are also reported by Netto et al. (2000) and Jackson & Langguth (1987). Other researchers have observed that exist, in cervidae species, an important relationship between the group size, habitat conditions and feeding style and has been suggested that the differences are not only among species, but also may be among populations; and individuals, which may adopt the appropriate structure for their habitat (Putman, 1988;Putman & Flueck, 2011).

Several authors have considered the pampas deer as a “concentrate selectors” (Pinder, 1997;Rodrigues & Monteiro-Filho, 1999), or “mixed grass feeder” (Jackson & Giulietti, 1988;Cosse, González et al., 2009) according to Hofmann classification (Hofmann, 1989). The possible explanation to the recorded differences in feeding strategies may be also due to the phytogeographical variation throughout the pampas deer’s range. The nutritional value of Uruguayan and Argentinean temperate grasslands (C3) are higher than the rough tropical (C4) vegetation dominant in the Cerrado, which exhibit high dry weight accumulations that are often of low nutritive value (Van Soest, 1994). In the cerrado of the Pantanal the phenological succession correspond with rainy, flood and dry seasons (Pinder, 1997). It will be interesting to obtain more data on TGS for different pampas deer populations to assess on the change on this variable related to the nutritional quality of the pastures.

Other factor that influences the grouping size is related with reproductive behavior. The female segregation observed and the reduction in the size of nursery groups around the birth season has explained changes in the median group size. Meanwhile, in the mating season we observed in Los Ajos population a change in the conformation of the mixed-sex groups with larger proportions of this kind of groups. Similar observations were performed by Lacerda (2008).

Another analyzed factor was the available pasture in areas shared with livestock. For mixed-sex groups our data showed the major size on feeding groups was on winter in which was available ray grass pasture for cattle.

Thus, based on our results, pampas deer groups’ structure and dynamics depend both on seasonal variation and in the requirements of individuals based on their sex and reproductive status. Then, in this population individuals associations pattern are link with the individual’s optimal efficiency (fitness) (Krause & Ruxton, 2002).

### Sexual aggregation-segregation

Several authors propose that segregation between the sexes occurs in sexually dimorphic species because the difference in body size between adult males and adult females entails different feeding needs, “forage-selection hypotheses” (Clutton-Brock et al., 1982;Illius & Gordon, 1987, Ruckstuhl, 1998;2000;Yearsley & Javier Pérez-Barbería, 2005). We observed a sexual aggregation-segregation pattern limited to a very specific reproductive and physiological moment (Figure 3). Pampas deer exhibits a low level of polygyny (0.61 ♂:♀), and little sexual dimorphism in body size, the body mass of adult males being approximately 1.17 times that of adult females (González et al., 2010). The existence of a pattern of spatial segregation associated with sexual segregation could be linked to the “predation risk hypothesis” (Ruckstuhl & Neuhaus, 2000), with females with small fawns staying in safer habitats while males choose for better quality pastures to meet their nutritional requirements during antler development, but it is necessary to deeper analysis on spatial segregation and pasture quality in order to have more categorical conclusions.

### Conservation implications

Pampas deer in Los Ajos occur exclusively in agro-ecosystems within several ranches on a RAMSAR Biosphere Reserve. This remaining population of the pampas deer has a critical size, which showed significant variations along the years. From a management perspective the PVA showed the populations has the intrinsic potential to survive being important to assure and increase the habitat available to guarantee the expansion and persistence in the future years.
